# Asymmetric evidence of foreign direct investment response to stock returns in Nigeria

**DOI:** 10.1186/s43093-023-00194-4

**Published:** 2023-05-13

**Authors:** Joel Ede Owuru, Olumuyiwa Samuel Oladele

**Affiliations:** 1 Department of Economics, Faculty of Humanities, Managements and Social Sciences, Augustine University, Ilara-Epe, Lagos State, Nigeria; 2 Department of Accounting and Finance, Faculty of Humanities, Managements and Social Sciences, Augustine University, Ilara-Epe, Lagos State, Nigeria

**Keywords:** Foreign direct investment, Stock returns, Nonlinear autoregressive distributed lag, C50, E32, F21, G11

## Abstract

This study examines the response of foreign direct investment (FDI) to asymmetric changes in stock returns in Nigeria. We employed disaggregated monthly data for Nigeria (1985M01–2017M12) to test the magnitude of stock returns’ elasticity of FDI using a nonlinear ARDL modelling framework. By controlling for exchange rate and output size, evidence of asymmetric response of FDI to stock market returns in Nigeria could not be denied. Hence, sustainable development in Nigeria requires a long-run balance between FDI and Stock market through a stable exchange rate management system and efficiency of the capital market is necessary for increased FDI.

## Introduction

Foreign Direct Investment (FDI hereafter) is a key globalizing factor that has increasingly integrated different economies of the world. Investments in domestic economies by investors from foreign countries with full direct control of ownership of such businesses are dubbed as FDI, and they are unlike foreign portfolio investment (FPI) where investors do not have full direct control of ownership. Morisset [15] asserts that FDI involves injection of foreign funds into domestic economy through enterprises that are resident in that economy differently from the country of origin of investors. In FDI management, foreign investors are granted management and voting rights if the level of ownership is greater than or equal to 10% of ordinary shares, hence, any shares ownership less than this threshold is rather referred to as FPI [3, 15].

OECD [22] extensively details the structural forms of FDI and their numerous merits, it provides stable and durable linkages between the home (foreign) and the host economies (domestic) that lead to development. Also, the United Nation Conference on Trade and Development, UNCTAD [44], in its World Investment Report asserts, in congruence with OECD [21] and IMF [14] that FDI involves a long-term relationship and reflects a lasting interest and control by a resident entity in one economy (foreign direct investor or parent enterprise) in an enterprise resident in an economy other than that of the foreign direct investor (FDI enterprise or affiliate enterprise or foreign affiliate). Such home-host countries’ relationship through FDI often create enabling environment for technology transfer, and development of local enterprises, and employment generation. As good numbers of Multinational Companies (MNCs) are expanding abroad in view of easy access to raw materials, it is expected that gains from such expansions should not be lopsidedly distributed, it should be mutually bilateral such that as raw materials for business expansions are obtained from the host economies of investors, the profit should not be totally repatriated to the home economies of investors without allowing it trickle down and boost the level of growth and sustainable development in the home economies.

**Fig. 1:**
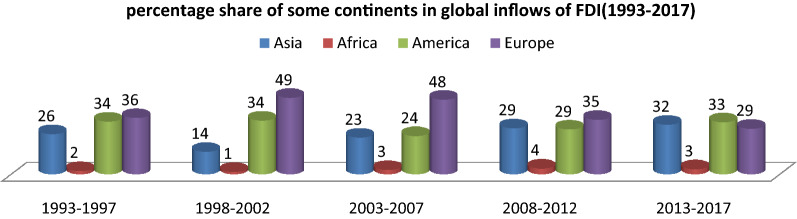
Periodic continental shares of Global FDI inflows. *Source*: Authors’ computation from UNCTAD database

It is shown in recent times that the trend of global inflow of FDI favours developing countries. UNCTAD [43] reports that interest in developed countries as destination of FDI compared to other regions has declined over the past few years and is likely to continue in the near future. Thus, we find in Fig. 1 that the developing countries including Asian tigers and Latin American among others received higher percentage share of global inflows of FDI.

Notwithstanding this dismal performance of Africa in attracting high global FDI inflow based on the periodic stylized fact in Fig. 1, recent evidence holds that for the third year in a row, FDI is down all over the world, but not in Africa [42]. This source further articulates in its World Investment Report that from 2017 to 2018, global FDI fell from $1.5 trillion to $1.3 trillion, and that FDI not only hit its lowest level since the global financial crisis, but has also been on the decline for three consecutive years with exception of Africa that recorded roughly $46 billion worth of FDI inflows. This represents 11% increase compared to 2017. It was further believed that the signing into law of the recent agreement of African Continental Free Trade Area (AfCFTA) promotes these inflows [42].

**Fig. 2:**
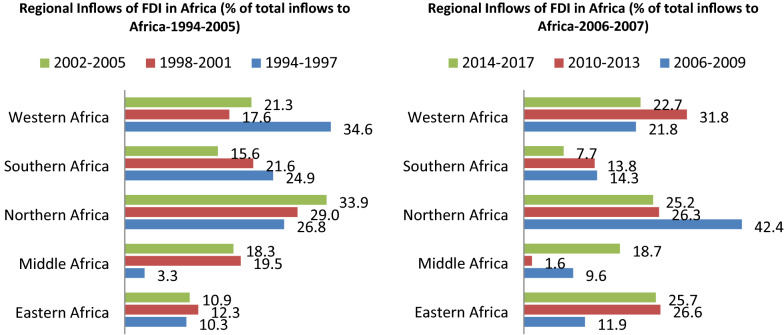
Regional shares of global FDI inflows to Africa. *Source*: Author’s Computation from [42]

In these recent upsurges in FDI inflow to Africa, West Africa sub-region attracts larger shares. Between 1994 and 2005, Western Africa attracted approximately 35% of global FDI inflow to Africa. Northern Africa (Egypt in particular) attracted 34% (see Fig. 2 for evidence). Between 2006 and 2017 however, Northern Africa got 42.4% while Western Africa attracted 32%.

It is obvious that economies in Western Africa, especially Nigeria occupy a central space in attracting global FDI inflow to the continent. This is not unconnected with availability of abundance resources in this region particularly oil and gas sector of Nigeria. Other economies in this region such as Ghana and Côte d'Ivoire also attract relatively high percentage of total global FDI inflows to Western Africa (see Table 1 for evidence).

**Table 1:** Periodic percentage shares of Total FDI inflows to Western Africa (1994–2017)

Country	1994–1997	1998–2001	2002–2005	2006–2009	2010–2013	2014–2017
Benin	0.5	1.9	1	1.4	1.6	1.9
Burkina Faso	0.5	0.5	0.5	1.3	1.6	3.2
Cabo Verde (Cape Verde formerly)	0.7	1.4	1.3	1.6	0.8	1.1
Côte d'Ivoire	10.2	16.6	5.7	3.6	2.2	4.7
Gambia	0.6	1.7	1	0.6	0.2	0.2
Ghana	5.4	6.8	2.7	12.8	19.7	28.7
Guinea	0.4	1	1.9	2.1	2.9	5
Guinea-Bissau	0.1	0.1	0.1	0.1	0.1	0.2
Liberia	1	5.3	3.1	1.7	5.3	3.5
Mali	2.4	2.4	4.1	2.5	2.7	2.2
Niger	0.2	0.3	0.4	3	5.7	4.3
Nigeria	74.1	55	66.3	63.8	44.5	33.9
Senegal	2.8	3.4	1.5	2.8	1.9	3.9
Sierra Leone	0.1	0.5	1	0.7	3.8	2.9
Togo	0.8	1.7	1.3	0.5	1.8	0.9

*Source*: Author’s Computation from [42]

**Fig. 3:**
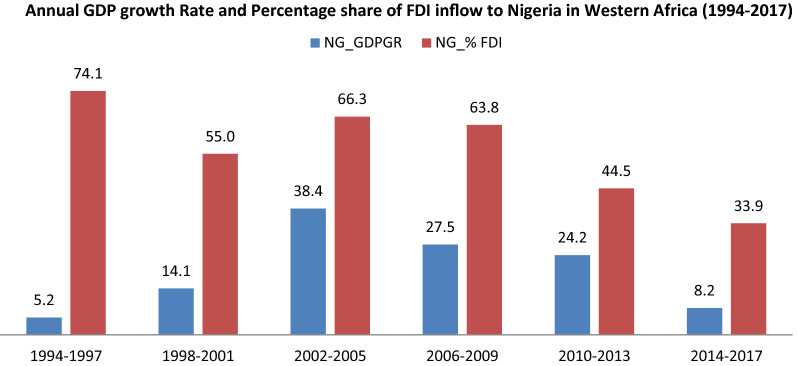
Annual GDP growth rate and FDI shares of Nigeria in total inflow to western Africa. *Source*: Author’s Computation from UNCTAD [42, 47]

Despite the revelations from the foregoing discussions that Nigeria consistently outperformed other economies in Western Africa in FDI inflows, there exists apprehensions from various circles (academics, policy analysts, stakeholders or/and civil societies) that the trend of development proceeds from output growth in Nigeria is not sustainable and inclusive. It could be seen from Fig. 3 that the trend of annual growth rate of the Gross Domestic Product (GDP) of Nigeria is not in tandem with that of FDI inflows for the fiscal period (1994–2017). The implication of this development is that FDI-led impacts have not sustainably addressed developmental challenges in Nigeria (the host country). This could be caused, either by failed/inappropriate fiscal policy responses of Nigeria to benefit from FDI's proceeds or the foreign investors are not fully committed to their statutory corporate social responsibilities (CSR) in its host economy.

The main motivation for this study lies in the area of FDI-Stock market nexus in Nigeria. We arguably assert that as FDI are injected into the domestic economy, it goes through the financial market and largely translated to the capital market. Again, there is possibility of re-investing plough back profits stocks. It was recently observed that not too long from the period that a Multinational mobile telecommunication company based in South Africa with operations in different African countries, called Mobile Telephone Network (MTN) was listed in the Nigerian stock exchange recorded some losses depending on the volatility of stock price returns like other firms. Thus, there is a seeming reality that the level of efficiency of the capital market structure and its asymmetric returns could determine subsequent FDI inflows. However, we surprisingly observed that there is dearth of empirical studies that unearth the linkage of FDI with stock market returns.

Most of the available studies dwell on three stances. The first examines impacts of FDI on stock market development in Nigeria (see [20, 29, 30] among others) while the second stance relates to studies on the effects of FDI on economic growth (see [13, 24, 41, 45, 46] among others for Nigeria, and [4] for a global review of FDI effects in various countries output between 1994 and 2012), the third group includes studies that examine the effects of Stock or capital market development on economic growth (see [18], Osinubi and Amaghionyeodiwe [31]; Adamu and Sanni [2]; [1, 7−10. 19, 23, 26−28, 34] among others for Nigeria and [25] for Stock market-Economic growth nexus for other developing countries).

To the best of our knowledge, extant literature on the impact of stock market outcomes on FDI in Nigeria is scare. The only notable study that partially relate to our investigation is Ezeoha et al. [11] who examined the impact of stock market development on the level of investments (Domestic Private Investment and Foreign Private Investment). This study however failed to account for asymmetries in the analysis of FDI response to stock market development. It is even a handful of international empirical studies that exist in this regard (see [6, 35] for example). Indeed, FDI could respond to financial market conditions in the host economies of such investment and therefore serves as domestic absorptive capacity that can boost the impacts of FDI inflows to host economies. Our study therefore contributes to knowledge in stock-led FDI response in developing economies with evidence from Nigeria.

To fill this identified lacuna in the literature on FDI-Stock market nexus, this study beams it searchlight on the direction of asymmetric response of FDI to stock market returns in Nigeria by exploring the monthly time series of Nigeria stock prices and FDI inflows from 1985M01 to 2017M12. Through these innovations, this study provides answers to key empirical questions such as: do shocks to stock market returns in Nigeria asymmetrically matter for FDI inflows to the country? Although we recognize the fact, based on evidence that the COVID-19 pandemic has impacted greatly the financial markets across the globe with attendant consequences on FDI through international market risk exposures, this study is however, limited to the pre-Pandemic periods and does not cover the volatile era of the pandemic.

In order to provide empirical answers to this question, we laid the structure of the residual of this paper as follow: the methodology of the study is detailed in Sect. “Methodology and data”. While Sect. “Empirical Results” presents the empirical results and the discussion of the findings with relevant policy implications, Sect. “Concluding Remarks” concludes the study.

## Methodology and data

### Methodology and model specification

In modelling the magnitude of asymmetric response of FDI to Stock market returns in Nigeria, we adopts the recently invented model of Nonlinear Autoregressive Distributed Lag (NARDL) by Shin et al. [40] in examining the short-run and long-run asymmetrical effects of stock market returns on FDI in Nigeria. This NARDL model is important because sudden shocks or changes in the series could cause volatile behaviour of the series, and in fact, failing to account for changes in FDI series in relation to asymmetric dynamics of stock returns when it actually exist can render empirical results and its attendant policy prescription bias (see [12, 16, 36, 37]). The advantage of this modelling innovation with NARDL in this study is related to the various merits of NARDL approach detailed by Nusair [17].

The functional form of the model for this study is stated in Eq. (1).

1 $$LnFDI = f(LnGDP,LnEXR,LnASI)$$

$LnFDI{\text{, LnGDP, LnEXR,}}$ and ${\text{LnASI }}$ represent the natural logarithmic values of FDI, Gross Domestic Product (GDP), Exchange rate, and Al price index of stock price. Equation (1) shows that the variables in the R.H.S of Eq. (1) are exogenously determined and their effects will be estimated on FDI inflows to Nigeria. Stock market returns is calculated from LnASI as ${STR}_{t}$ = 100* [Δ Ln (${ASI}_{t}$)]^1^ where ASI is the all share index of stock prices. Having established that FDI can respond differently to stock market returns, the series is decomposed into positive and negative returns following the NARDL framework of Shin et al. [40]. In this case, the asymmetric returns of stock prices are decomposed to account for short-run and long-run asymmetries in stock returns. Therefore, the partial sum decomposition of the positive and negative stock market returns series is structured in Eqs. 2 and 3, respectively.

2 $$STR_{t}^{ + } = \sum\limits_{j = 1}^{t} {\Delta STR_{j}^{ + } } = \sum\limits_{j = 1}^{t} {\max (\Delta STR_{j} ,0)}$$

3 $$STR_{t}^{ + } = \sum\limits_{j = 1}^{t} {\Delta STR_{j}^{ + } } = \sum\limits_{j = 1}^{t} {\min (\Delta STR_{j} ,0)}$$

The positive or negative returns in stock market returns in Eqs. (2) and (3) represent return gain or loss in stock market to the investors. Thus, by utilizing these decomposed asymmetric changes in the stock price series in the fashion of Shin et al. [40], the estimated NARDL model for this study is specified in Eq. (4) as:

4 $$\begin{aligned} \Delta LnFDI_{t} & = \alpha_{0} + \sum\limits_{i = 1}^{p} {\beta_{1} \Delta LnFDI_{t - i} + } \sum\limits_{i = 1}^{p} {\beta_{2} \Delta LnGDP_{t - i} + \sum\limits_{i = 1}^{p} {\beta_{3} \Delta LnEXR_{t - i} + } } \sum\limits_{i = 0}^{q} {(\beta_{4}^{ + } \Delta STR_{t - 1}^{ + } + \beta_{4}^{ - } \Delta STR_{t - 1}^{ - } )} \\ & \quad + \alpha_{1} LnFDI_{t - 1} + \alpha_{2} LnGDP_{t - 1} + \alpha_{3} LnEXR_{t - 1} + \alpha_{4}^{ + } STR_{t - 1}^{ + } + \alpha_{4}^{ - } STR_{t - 1}^{ - } + \varepsilon_{t} \\ \end{aligned}$$

where the variables with a sign of differential change ($\Delta$) denotes the short the short-run parameters. The NARDL model in Eq. (4) can therefore be re-specified in form of short-run error correction model as:

5 $$\begin{aligned} \Delta LnFDI_{t} & = \sum\limits_{i = 1}^{p} {\beta_{1} \Delta LnFDI_{t - i} + } \sum\limits_{i = 1}^{p} {\beta_{2} \Delta LnGDP_{t - i} + \sum\limits_{i = 1}^{p} {\beta_{3} \Delta LnEXR_{t - i} + } } \sum\limits_{i = 0}^{q} {(\beta_{4}^{ + } \Delta STR_{t - 1}^{ + } + \beta_{4}^{ - } \Delta STR_{t - 1}^{ - } )} \\ & \quad + \lambda ECM_{t - 1} + \mu_{t} \\ \end{aligned}$$

where $ECM_{t - 1} = LnFDI_{t - 1} - \theta^{ + } STR_{t - 1}^{ + } - \theta^{ - } STR_{t - 1}^{ - }$ is the nonlinear error correction term with $\lambda$ as the speed of the short-run adjustment to long-run equilibrium, the long-run parameters of the model in Eq. (4) are defined as $\theta^{ + } = - \frac{{\alpha_{4}^{ + } }}{{\alpha_{1} }}$ and $\theta^{ - } = - \frac{{\alpha_{4}^{ - } }}{{\alpha_{1} }}$ while the associated coefficient for measuring short-run adjustment of positive and negative changes in stock returns are captured by $\beta_{4}^{ + }$ and $\beta_{4}^{ - }$ , respectively.

### Model estimation techniques

To estimate the specified NARDL model, we first run the bound testing approach to cointegration developed by Pesaran et al. [32] is applied to test for the presence of long-run relationship between the variables. This method of cointegration has superior advantages over the classical methods. Principally, if the series are not stationary in the same order, cointegration among them can still be tested [32]. By applying the bound test that follows F-distribution to the specified models above, the null hypothesis of no cointegration ($H_{0} :\alpha_{1} = \alpha_{4}^{ + } = \alpha_{4}^{ - } = 0$) is tested against its alternative hypothesis of the existence of cointegration ($H_{0} :\alpha_{1} \ne \alpha_{4}^{ + } \ne \alpha_{4}^{ - } \ne 0$).

To test for the presence of asymmetries in the long-run or otherwise, the standard Wald test is estimated to test the null hypothesis of no asymmetries in the long-run ($H_{0} :\alpha_{4}^{ + } = \alpha_{4}^{ - } = 0$) against its alternative hypothesis of the presence of asymmetries ($H_{0} :\alpha_{4}^{ + } \ne \alpha_{4}^{ - } \ne 0$).

Similarly, for the short-run, presence or otherwise of asymmetry is tested with the null hypothesis of no asymmetries $\left( {H_{0} :\sum\limits_{i = 0}^{q} {\beta_{4}^{ + } = \sum\limits_{i = 0}^{q} {\beta_{4}^{ - } = 0} } } \right)$ as against its alternative of the presence of asymmetries

$$\left( {H_{0} :\sum\limits_{i = 0}^{q} {\beta_{4}^{ + } \ne \sum\limits_{i = 0}^{q} {\beta_{4}^{ - } \ne 0} } } \right).$$

Asides bound test with its associated tests for asymmetries, other post-estimation diagnostic tests such as model stability, serial correlation, and heteroscedasticity test are carried out as robustness check to enhance the validity of any policy inference that would be drawn from the empirical results of this study.

### Variable description and data sources

This study covered the period of 1985 to 2017. The four variables covered in this study include Foreign Direct Investment (FDI), Gross Domestic Product (GDP), Exchange rate (EXR), and Stock prices. The data for these variables were obtained from The United Nation Conference on Trade and Development [42] online database and The World Bank Development Indicators [47]. The data were disaggregated into high frequency data (monthly) starting from January 1985 to December 2017.

## Empirical results

The estimated results for the specified model for this study are presented here. The section starts with the presentation of all the preliminary tests such as the descriptive statistics of the series, the trend of their behaviour overtime the study period, and the results from the unit root tests.

### The preliminary results

#### The summary statistics

The results in Table 2 show that all the variables have positive average values with natural logarithmic values of GDP as the highest. Stock returns are positive and such positive average returns are expected to have positive effects on investment in stocks. Again, from Table 2, all the variables are negatively skewed with the exception of negative stock return series. Such skewedness implies that there is extremity of fat tail to the left. For Kurtosis, the summary statistics revealed that all the variables, except stock returns which showed high peak (leptokurtic) were platykurtic in nature, and as such, they did not exhibit high peak as they fall below the threshold of 3. On the part of normality test of the distribution of the series, Jarque–Bera statistics showed that the data sets are not normally distributed. Lastly, from the measure of dispersion, the standard deviations of the series are not wide, implying that the successive series are not so much at variance with expected normal distribution at the course of data generation process.

**Table 2:** Summary statistics of the series

Statistics	LNFDI	LNGDP	LNEXR	Stock returns	Positive stock returns	Negative stock returns
Mean	7.68	29.60	3.81	1.48	5.32	3.28
Median	7.67	29.74	4.72	1.60	6.32	0.00
Maximum	9.12	32.44	5.74	33.00	11.08	11.05
Minimum	5.09	25.92	− 0.17	− 36.58	0.00	0.00
Std. dev	0.95	2.07	1.53	6.12	4.27	4.54
Skewness	− 0.51	− 0.34	− 0.89	− 0.55	− 0.23	0.72
Kurtosis	2.97	1.80	2.81	10.10	1.39	1.61
Jarque–Bera	17.33	31.23	53.01	850.57	46.15	66.07
Probability	0.00***	0.00***	0.00***	0.00***	0.00***	0.00***
Sum	3032.95	11,693.41	1506.45	583.95	2102.16	1295.34
Sum Sq. dev	354.05	1683.45	918.32	14,746.35	7173.54	8119.01
Observations	395	395	395	395	395	395

Source: Author’s computation from the underlying data

***Denotes 1% probability value

#### Trends of the series

**Fig. 4:**
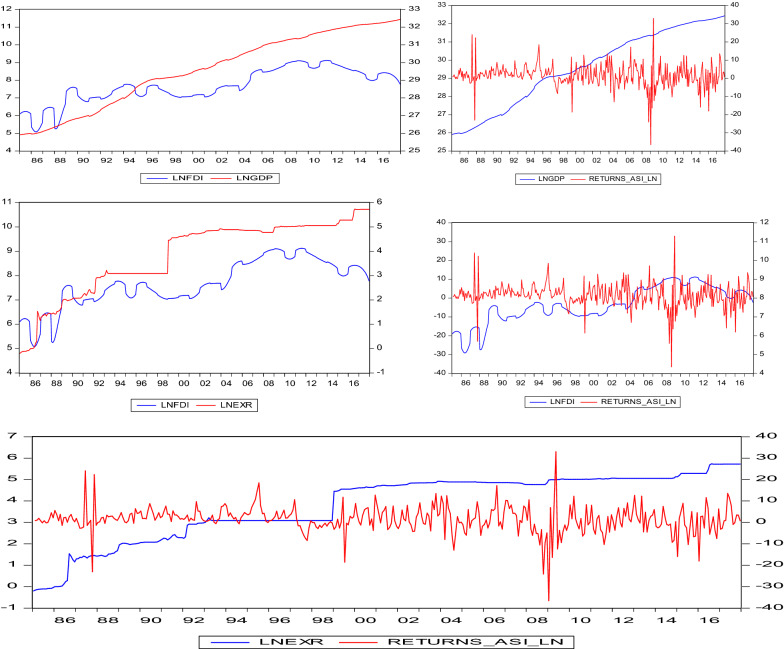
Trends of FDI, GDP, exchange rate, and stock market returns in Nigeria. *Source*: Author’s computation from the underlying data

In addition to the descriptive statistics shown in Table 2, the cyclical behaviour of the series indicates that there is structural instability causing the dynamics of FDI inflows to Nigeria to meander from period to period. Again, there is an indication of volatility clustering in stock market return series over the time covered in this study. In this case, periods of high volatility are followed by periods of low volatility (see Fig. 4).

#### Stationarity tests

To ensure that the estimated results are valid, a preliminary test of unit root was done. For robustness, two unit root tests were used, the augmented Dickey–Fuller (ADF) and the Phillip–Perron test to check for the order of stationarity of the variables. Table 3 presents the results for all the series for both tests. From the results, the series have a mixture order of integration as I(0) and I(1). Natural logarithmic value of FDI and GDP exhibit non-stationarity at level I(0) based on Phillips–Perron test. Thus, the choice of using Autoregressive Distributed lag (ARDL) as the series (altogether) are fractionally integration is justified in-line with the novel proposition of ARDL by Pesaran and Shin [33]and Nusair [17]. It is because of the need to examine the roles of asymmetries in the model that stock market return series was decomposed to account for nonlinearity [40], hence the decomposed series are also tested for order of their integration, and this underscored the choice of NARDL model as the main estimated model.

**Table 3:** Unit root tests

(a)												
Augmented Dickey–Fuller (ADF)							Phillips–Perron (PP)					
	Constant		Constant and trend		None		Constant		Constant and trend		None	
Variables	*t*-stat.	*P*-value	*t*-stat.	*P*-value	*t*-stat.	*P*-value	*t*-stat.	*P*-value	*t*-stat.	*P*-value	*t*-stat.	*P*-value
*Level*												
LNFDI	− 2.72	0.07*	− 1.44	0.85	1.12	0.93	− 2.06	0.26	− 2.54	0.31	0.28	0.77
LNGDP	− 2.64	0.09*	− 0.57	0.98	2.93	0.99	− 2.18	0.21	0.18	0.99	8.42	1.00
LNEXR	− 2.67	0.08*	− 2.62	0.27	1.71	0.98	− 2.72	0.07*	− 2.61	0.28	1.68	0.98
STR	− 6.75	0.00****	− 7.02	0.00***	− 6.14	0.00***	− 158.26	0.00***	− 17.87	0.00***	− 17.86	0.00*
STR(+)	− 15.36	0.00***	− 15.36	0.00***	− 2.12	0.03**	− 16.03	0.00***	− 16.03	0.00***	− 9.30	0.00*
STR(−)	− 9.51	0.00***	− 15.35	0.00***	− 3.02	0.00***	− 15.51	0.00***	− 15.84	0.00***	− 12.82	0.00*
*First difference*												
LNFDI	− 6.67	0.00***	− 7.05	0.00***	− 6.50	0.00***	− 16.08	0.00***	− 16.08	0.00***	− 16.09	0.00***
LNGDP	− 3.64	0.01***	− 4.45	0.00***	− 1.36	0.16	− 14.17	0.00***	− 14.57	0.00***	− 7.50	0.00***
LNEXR	− 19.54	0.00***	− 19.67	0.00***	− 19.16	0.00***	− 19.54	0.00***	− 19.68	0.00***	− 19.17	0.00***
STR	− 11.99	0.00***	− 11.98	0.00***	− 12.01	0.00***	− 158.26	0.00***	− 158.03	0.00***	− 158.57	0.00***
STR( +)	− 15.34	0.00***	− 15.32	0.00***	− 15.35	0.00***	− 113.36	0.00***	− 112.07	0.00***	− 112.98	0.00***
STR(−)	− 15.60	0.00***	− 15.58	0.00***	− 15.62	0.00***	− 103.54	0.00***	− 103.67	0.00***	− 103.72	0.00***

(b)						
	Augmented Dickey–Fuller (ADF)			Phillip–Perron (PP)		
	Level	First difference	Remark	Level	First difference	Remark
Variables	t-stat.	t-stat.	*I(d)*	*t*-stat.	*t*-stat.	*I(d)*
LNFDI	− 2.72^a^**	− 7.05^b^***	*I(0)*	–	− 16.08^b^***	*I(1)*
LNGDP	− 2.64^a^**	− 4.45^b^***	*I(0)*	–	− 14.57^b^***	*I(1)*
LNEXR	− 2.668^a^**	− 19.67^b^***	*I(0)*	− 2.72^a^**	− 19.68^b^***	*I(0)*
STR	− 7.02^b^***	− 11.98^b^***	*I(0)*	− 17.87^b^***	− 158.03^b^***	*I(0)*
STR( +)	− 15.36^b^***	− 15.32^b^***	*I(0)*	− 16.03^b^***	− 112.07^b^***	*I(0)*
STR(−)	− 15.35^b^***	− 15.58^b^***	*I(0)*	− 15.84^b^***	− 103.67^b^***	*I(0)*

(a) *, ** and ***Imply significance of the variables at 10%, 5%, and 1%, respectively

(b) ** and***Indicate significance level of the variable s at 5% and 1%, respectively. Any variable that is significant at both level and first difference is a level stationary series. Also, ‘a’ represents model with constant, while ‘b’ is for model with constant and trend

### The main results

#### NARDL bound test result

It is important to determine the possibility of the existence of long-run relationship (cointegration) among the variables. Thus, the bound test result for cointegration is examined in addition to the evidence from the stationarity test results above. Here, the null hypothesis of no cointegration is tested against its alternative of the presence of cointegration. Since the value of F-statistic is greater than the Critical Value Bounds for the upper bound *I(1)*, then the null hypothesis cannot be accepted. Therefore, there is cointegration implying the existence of a long-run relationship among the examined series. Based on this evidence of long-run cointegration in Table 4, FDI would react to host country’s economic size, exchange rate behaviour and asymmetric stock market returns in the long run if there are effective policy coordination in that direction.

**Table 4:** NARDL bound test result

Null hypothesis: No long-run relationships exist		
Test statistic	Value	K
*F*-statistic	14.75229	4
Critical value bounds		
Significance	I0 Bound	I1 Bound
10%	2.45	3.52
5%	2.86	4.01
2.5%	3.25	4.49
1%	3.74	5.06

Source: Author’s computation from the underlying data using Eview 9

#### Optimal lag length selection

The lag length helps in determining how many times (depending on the frequency of the data) backward in the autoregressive process (AR process) can the serial correlation of the series be tested. Thus, variables under consideration are tested to determine the best (optimal) of lag order selection using the conventional information criteria. From the results in Table 5, optimal lag length of the estimated series in the AR process is 5 based on Final prediction error (FPE), Akaike information criterion (AIC), and Hannan–Quinn information criterion (HQ).

**Table 5:** Optimal Lag structure of the series

Lag	LogL	LR	FPE	AIC	SC	HQ
0	− 2764.23	NA	1.311	14.46	14.51	14.48
1	418.17	6265.10	9.06e−08	− 2.03	− 1.72	− 1.90
2	498.48	155.99	6.79e−08	− 2.32	− 1.77*	− 2.09
3	536.45	72.78	6.35e−08	− 2.38	− 1.56	− 2.06
4	555.47	35.95	6.55e−08	− 2.35	− 1.27	− 1.92
5	685.90	243.15	3.78e−08*	− 2.90*	− 1.56	− 2.37*
6	707.29	39.31	3.85e−08	− 2.88	− 1.29	− 2.25
7	717.84	19.12	4.16e−08	− 2.81	− 0.95	− 2.07
8	729.17	20.23	4.47e−08	− 2.74	− 0.62	− 1.90
9	751.67	39.59*	4.54e−08	− 2.72	− 0.35	− 1.78
10	763.14	19.89	4.88e−08	− 2.65	− 0.02	− 1.61
11	772.62	16.20	5.31e−08	− 2.57	0.31	− 1.43
12	781.45	14.83	5.80e−08	− 2.49	0.66	− 1.24

*Indicates lag order selected by the criterion

*LR* sequential modified LR test statistic (each test at 5% level)

*FPE* Final prediction error

*AIC* Akaike information criterion

*SC* Schwarz information criterion

*HQ* Hannan–Quinn information criterion

#### NARDL cointegrating and long-run form

Having verified that there is long-run relationship among the variables from the bound test, the results from the estimated model shown in Table 6 reveal the magnitude of the elasticity of FDI (dependent variable) to each of its determinants in the NARDL model as well as the error correction term that shows the speed of convergence of short-run shocks to the long-run equilibrium. From the results in Table 6, it is glaring from the short-run model (upper side of the table) that there is significant short-run response of FDI to most of the explanatory variables such as changes in the current rate of exchange rate [D(LNEXR)], the third and the fourth lags of exchange rate-[D(LNEXR(− 3))] and D(LNEXR(− 4))], and both positive and negative stock returns-[D(STR^+^)] and [D(STR^−^)] among others. We also observed negative response of FDI to the first and second lagged and leads of FDI dynamics-[D(LNFDI(− 1), 2)] and [D(LNFDI(− 2), 2)]. Insignificant response to change in the second lead value of GDP [D(LNGDP, 2)], and the first and second lagged variables of change in exchange rate [D(LNEXR(− 1)) & D(LNEXR(− 2))].

**Table 6:** NARDL results

Variable	Coefficient	Std. error	*t*-statistic	Prob.
*Cointegrating Form (Short-Run Coefficients)*				
D(LNFDI(− 1), 2)	− 0.2384	0.0634	− 3.5858	0.0004***
D(LNFDI(− 2), 2)	− 0.0892	0.0499	− 1.7845	0.0751*
D(LNGDP, 2)	0.5315	0.3701	1.4361	0.1518
D(LNEXR)	0.0874	0.0499	1.7498	0.0810*
D(LNEXR(− 1))	0.0005	0.0703	0.0067	0.9946
D(LNEXR(− 2))	0.0013	0.0702	0.0189	0.9849
D(LNEXR(− 3))	− 0.2384	0.0739	− 3.2267	0.0014***
D(LNEXR(− 4))	0.2624	0.0577	4.5499	0.0000***
D(STR^+^)	0.0172	0.0081	2.1269	0.0341**
D(STR^−^)	0.0164	0.0079	2.0487	0.0412**
CointEq(− 1)	− 0.5752	0.0687	− 8.3750	0.0000***
Cointeq = D(LNFDI)—(0.9240*D(LNGDP) − 0.0299*LNEXR + 0.0298				
*STR^+^ + 0.0284* STR^−^− 0.1614)				
*Long-run coefficients*				
D(LNGDP)	0.9240	0.6443	1.4343	0.1523
LNEXR	− 0.0299	0.0176	− 1.6964	0.0906*
STR^+^	0.0298	0.0148	2.0202	0.0441**
STR^−^	0.0284	0.0145	1.9549	0.0513**
C	− 0.1614	0.0734	− 2.1988	0.0285**

Source: Author’s computation from the underlying data using Eview 9

*, **, and*** implies that the variable is significance at 10%, 5%, and 1%, respectively

From these results, any sudden short-run shocks to FDI significantly decreases subsequent inflows of FDI up to 0.23 per cent. Also, a 1 per cent increase in exchange rate (depreciation) will cause FDI inflow to increase by approximately 0.09 per cent in the short-run. This result shows a dynamic response in exchange rate elasticity of FDI in the short-run because devaluation of exchange rate, which can be assumed by third and fourth order of lag in exchange rate show different result. For instance, the third lag indicates significant inelastic response (decrease) of FDI to exchange rate by 0.24 per cent, while it responded significantly but elastically to the fourth lag of change in exchange rate by 0.26 per cent. These dynamic responses of FDI to exchange rate calls for policy action to stabilize exchange rate fluctuations in Nigeria to increasingly attract larger inflows of FDI.

Regarding FDI response to domestic output in the short-run, the result shows positive but insignificant relationship. In this case, GDP of the host economy may not matter most for going abroad decision of most foreign investors in the short-run. On asymmetric response of FDI to stock market returns in the short-run, we find that the significant responses of FDI to both positive and negative change in stock market returns in Nigeria are closely related. Here, the magnitude of positive and negative stock returns elasticity of FDI are 0.017 and 0.016 per cent, respectively. This implies that changes in domestic stock market returns could significantly pre-condition subsequent inflow of FDI to a given economy.

From the long-run structure of the results in the lower part of the result in Table 6, exchange rate, positive and negative shocks in stock market returns are also significant in determining long-term FDI inflows. While stock market asymmetric returns have almost the same magnitude of elasticity of FDI at 0.029 and 0.028, respectively, for positive and negative shocks, the response of FDI to exchange rate in the long-run is negative, but significant. Thus, depreciation in exchange rate will potentially diminish FDI inflows and rather enhance domestic private investment and boost domestic industrial capacity building in order to reduce over dependency on foreign investors.

Overall, both the short-run and long-run model are important as the result for the error correction term [CointEq(− 1)] is statistically significant at 1 per cent with expected negative sign. Thus, with a coefficient of − 0.575181, it shows that any disequilibrium in the short-run in the series will speedily converged at an approximate rate of 58 per cent. These findings of positive impacts of stock returns on FDI corroborate with the empirical evidence obtained in Sri Lanka by Choong et al. [5] though it is contrary to the finding of Ezeoha et al. [11] for Nigeria where stock market development encourages domestic private investment flows but failed to promote FDI in Nigeria.

#### Wald test results for asymmetries

Having found from the NARDL results that FDI respond positively and significantly to both positive and negative shocks in stock returns, it is important to validate if asymmetries actually matters in FDI-stock returns nexus to guide macroeconomic policy decision in Nigeria. Thus, the Wald standard test of coefficient restriction is applied and the results are contained in Table 7. Since the associated *P*-value of F-distribution of Wald test are greater than 0.1 (10%), the null hypothesis of no asymmetries is failed to be rejected, hence there is existence of asymmetries in FD response to stock market outcomes in Nigeria. By implication, foreign investors can respond differently to stock market outcomes in their host countries.

**Table 7:** Asymmetric test result

Wald test:			
Equation: Untitled			
Test Statistic	Value	Df	Probability
F-statistic	2.331482	(2, 378)	0.0985*
Chi-square	4.662964	2	0.0972*
Null Hypothesis: C(11) = C(12) = 0			
Null Hypothesis Summary:			
Normalized Restriction (= 0)	Value	Std. Err	
C(11)	0.017168	0.008072	
C(12)	0.016350	0.007981	
Restrictions are linear in coefficients			

Source: Author’s computation from the underlying data using Eview 9

*10% probability value

#### Granger causality result

To be able to examine the direction of causality among the variables, a pairwise granger causality test result is presented in Table 8. From the results, uni-directional causality was found running from exchange rate to FDI, exchange rate to GDP, positive stock market returns to GDP, exchange rate to negative stock market returns, and FDI to negative stock market returns. By implication, exchange rate dynamics granger causes changes in FDI inflows and also capable of determining negative returns in Nigerian stock prices. Again, exchange rate can cause output shocks (GDP), and positive returns to stock prices will also propel increase in GDP.

**Table 8:** Granger causality test results

Null hypothesis:	Obs	*F*-Statistic	Prob
LNGDP does not Granger Cause LNFDI	394	1.6925	0.1854
LNFDI does not Granger Cause LNGDP	1.0658	0.3454	
LNEXR does not Granger Cause LNFDI	394	2.6411	0.0726*
LNFDI does not Granger Cause LNEXR	0.9589	0.3843	
Positive Stock Returns does not Granger Cause LNFDI	393	0.1956	0.8224
LNFDI does not Granger Cause Positive Stock Returns	1.1158	0.3287	
Negative Stock Returns does not Granger Cause LNFDI	393	0.1167	0.8899
LNFDI does not Granger Cause Negative Stock Returns	7.4771	0.0007***	
LNEXR does not Granger Cause LNGDP	394	13.0592	0.0003***
LNGDP does not Granger Cause LNEXR	1.3371	0.2638	
Positive Stock Returns does not Granger Cause LNGDP	393	2.8152	0.0611*
LNGDP does not Granger Cause Positive Stock Returns	1.3481	0.2609	
Negative Stock Returns does not Granger Cause LNGDP	393	2.3928	0.0927*
LNGDP does not Granger Cause Negative Stock Returns	11.8787	0.0001***	
Positive Stock Returns does not Granger Cause LNEXR	393	0.5174	0.5965
LNEXR does not Granger Cause Positive Stock Returns	0.9179	0.4002	
Negative Stock Returns does not Granger Cause LNEXR	393	0.5507	0.5770
LNEXR does not Granger Cause Negative Stock Returns	7.4843	0.0006***	

Source: Author’s computation from the underlying data using Eview 9

*, and***Denote that the variables are significant at 10% and 1%, respectively

Undoubtedly, if FDI inflows are misdirected, they can cause negative returns in stock prices. The significant results of the Granger causality evidence of uni-directional causality running from FDI to negative stock returns reveals absence of bidirectional or reverse causalities between FDI and Financial market (stock returns). By implication, FDI could cause negative stock returns.^2^ Notwithstanding this evidence from this study, it is would not be unrealistic that financial market outcome could substantially react to dynamic changes in FDI as well. This underscores the motivation of this study because FDI could largely come through various channels including the financial markets, affected by the intervening function of exchange rate and then trickle down to affect output. Table 8 shows further that while bidirectional causality was found between negative stock returns and GDP, no causality existed between GDP and FDI. Positive stock market returns bidirectionally granger causes FDI as well, and such two-way causality also existed between exchange rate and positive stock market returns.

### Model post-estimation tests

It is still necessary to test the validity of the estimated asymmetric model further for robustness sake. The diagnostic tests employed here are the structural stability test with CUSUM and CUSUM sum of square, serial correlation and heteroscedasticity. They help re-validate the robustness of the estimation include are carried out.

#### Residual stability test: CUSUM and CUSUM squares

**Fig. 5:**
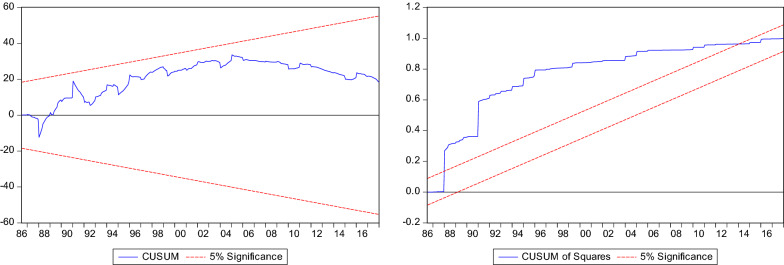
Cusum and cusum of square tests for model structural stability. *Source*: Author’s computation from the underlying data using Eview 9

The stability test for the estimated model using CUSUM and CUSUM of square is shown in Fig. 5. The plots showcase that the parameters of the model are relatively stable as the plot for CUSUM lies within the critical boundary. However, the CUSUM square plot reveals that there is no stability of the parameters; hence, there is high possibility of the variables being affected by structural breaks.^3^

#### Breusch–Godfrey serial correlation LM test

To check if the specified estimated model suffers from autocorrelation problem, the Breusch–Godfrey LM test is used to ascertain the validity or otherwise of the estimates. The null hypothesis of the test is that there is no serial correlation in the residuals up to the specified lag order. Since the probability values of the *F*-statistics is greater than 0.1 (maximum of 10% level of significance), the null hypothesis is not rejected. Thus, the estimates are valid and are not suffering from autocorrelation problem (see Table 9).

#### Heteroscedasticity test

The result for the Breusch–Pagan–Godfrey test for heteroscedasticity is shown in Table 10. The null hypothesis of this test is that there is no heteroscedasticity, and since the probability values of the *F*-test are greater than 0.01, 0.05, and 0.1 for 1%, 5%, and 10% (the three conventional levels of statistical significance), the null stands accepted, hence there is no heteroscedasticity problem in the estimated model of this study (see Table 9).

**Table 9:** Breusch–Godfrey Serial Correlation LM Test

Breusch-Godfrey Serial Correlation LM Test:			
F-statistic	0.959835	Prob. F(2,376)	0.3839
Obs*R-squared	1.986113	Prob. Chi-Square(2)	0.3704

Source: Author’s computation from the underlying data using Eview 9

**Table 10:** Breusch–Pagan–Godfrey heteroscedasticity Test

Heteroscedasticity Test: Breusch–Pagan–Godfrey			
F-statistic	1.099030	Prob. F(12,378)	0.3594
Obs*R-squared	13.18201	Prob. Chi-Square(12)	0.3560
Scaled explained SS	350.0283	Prob. Chi-Square(12)	0.0000***

Source: Author’s computation from the underlying data using Eview 9

Note: *** denotes that the measured statistic is significant at 1% level of statistical significance

#### Results discussion and policy implication

The empirical results obtained from this study have a number of implications for policy in Nigeria. In the international business cycle, FDI is instrumental in shaping the dynamics of interactions of many economies in the world. Its inflow to the host economy is an injection or a stimulus to economic growth. However, as FDI flows in, the financial sector is the medium through which such funds get transmitted into the economy and are invested in capital market (stocks) in different portfolios. Therefore, sustainable development in Nigeria requires a long-run balance between FDI and Stock market through a stable exchange rate management system. In this connection, this study finds that there is no causality between FDI and stock market returns even though FDI inflows proves to respond positively to asymmetric changes in stock returns in Nigeria. This therefore confirms that there is a dynamic interaction in FDI-Stock return nexus in Nigeria.

Proving from the empirical evidence that stock return elasticity of FDI is asymmetrical, it glaring that investors may have various goals in mind for going abroad. That is either improved or low stock returns still attract foreign investors. Irrespective of their business objectives, there is need for tight regulation of such foreign investors through appropriate tax policy such as pollution taxes so that the host economy will not be endangered environmentally. Also, the proceeds from such FDI and associated taxes could be used to strength needed sectors in domestic economy. Based on the results as well, FDI respond to negative stock returns. The policy lesson therefore is that the local investors should also be encouraged to invest in stocks.

Exchange rate also plays a vital role in determining the level of inflows of FDI. As results, the policy managers of the Nigerian economy should work on how to stabilize the rate of naira depreciation in connection with her trading partners. If such policies are implemented appropriately, FDI can be utilized to develop the economy of Nigeria.

There is also a strong connection between exchange rate, GDP and stock market returns which boils down to the fact that if there are viable policy-mix directed at addressing the volatility of exchange rate in Nigeria, there will be sustainable level of growth and development as business will begin to boom.

## Concluding remarks

The potential of stock market returns in predicting inflow of foreign investment in Nigeria is identified in this study as one of the necessary preconditions or domestic absorptive capacity of the home economy’s capital market to attract larger percentage of FDI inflows into the country. By employing a NARDL estimation technique on Nigerian data over two decades, a monthly data of, the study showed, among other things that the impact of stock returns asymmetries matter for FDI inflows in Nigeria. Again, with the controlled variables, the results revealed that asymmetric stock return pass-through to FDI inflows comprises the impact of exchange rate and economic size (GDP) on FDI as well, of which policymakers should be aware on how to target exchange rate stability for increasing output stabilization and sustainable growth. These findings of positive impacts of stock returns on FDI corroborate with the empirical evidence obtained in Sri Lanka by Choong et al. [5] though it is contrary to the finding of Ezeoha et al. [11] for Nigeria where stock market development encourages domestic private investment flows but failed to promote FDI in Nigeria. Efficiency capital market development in Nigeria is therefore important for increased FDI inflow to Nigeria.

## Data Availability

The data used for this research were sourced from the United Nations Conference on Trade and Development-UNCTAD https://unctadstat.unctad.org/EN/ and the World Development Indicators-WDI of the World Bank https://databank.worldbank.org/source/world-development-indicators.

## Abbreviations

ARDL: Autoregressive distributed lag
FDI: Foreign direct investment
GDP: Gross domestic product
IMF: International monetary fund
MNCs: Multinational companies
NARDL: Nonlinear autoregressive distributed lag
OECD: Organisation for economic cooperation and development
UNCTAD: United Nations Conference on Trade and Development
WDI: World Development Indicators

## Footnotes

1 See Salisu and Isah [38]   and Salisu et al. [39].

2 Although this is not the focus of our study as we only examine FDI response to financial market outcome. However, some situations could warrant FDI causing negative stock returns, especially when FDI crowd-out domestic investment. Here, local or domestic investors may start having marginal propensity to invest locally due to overwhelming effects of FDI in such economies. Also, any political or institutional policies in the home countries of foreign investors may have effects on the host economies; hence FDI could cause such negative financial market outcome.

3 Subsequent studies could examine structural breaks implications for FDI-Stock market nexus.
